# Changes in systemic medication in children and adolescents with JIA: why, when and which patterns

**DOI:** 10.1093/rheumatology/keag296

**Published:** 2026-06-10

**Authors:** Merel Boer-de Boer, Sytze de Roock, Toine C G Egberts, Arief Lalmohamed, Joost F Swart

**Affiliations:** Department of Clinical Pharmacy, University Medical Center Utrecht, Utrecht, The Netherlands; Division of Pediatrics, Department of Pediatric Rheumatology, Wilhelmina Children’s Hospital, University Medical Center Utrecht, Utrecht, The Netherlands; Faculty of Medicine, Utrecht University, Utrecht, The Netherlands; Department of Clinical Pharmacy, University Medical Center Utrecht, Utrecht, The Netherlands; Division of Pharmacoepidemiology and Clinical Pharmacology, Utrecht Institute for Pharmaceutical Science, Faculty of Science, Utrecht University, Utrecht, The Netherlands; Department of Clinical Pharmacy, University Medical Center Utrecht, Utrecht, The Netherlands; Division of Pharmacoepidemiology and Clinical Pharmacology, Utrecht Institute for Pharmaceutical Science, Faculty of Science, Utrecht University, Utrecht, The Netherlands; Division of Pediatrics, Department of Pediatric Rheumatology, Wilhelmina Children’s Hospital, University Medical Center Utrecht, Utrecht, The Netherlands; Faculty of Medicine, Utrecht University, Utrecht, The Netherlands

**Keywords:** JIA, MTX, bDMARDs, medication change, pharmacoepidemiology, paediatric rheumatology

## Abstract

**Objective:**

To investigate why, when and in which patterns decisions about changes in systemic medications were made in a cohort of JIA patients.

**Methods:**

JIA patients starting first-line systemic therapy were prospectively followed in a Dutch tertiary paediatric rheumatology centre. Treatment lines were constructed for the included systemic therapies (systemic corticosteroids, conventional and biological DMARDs (cDMARDs and bDMARDs)). Each change in systemic medication and its reason was registered. Main outcomes were frequencies and timing of change reasons, across treatment lines. A Sankey diagram was used to visualize flows between change reasons over treatment lines, cumulative incidences Fine-Gray models to illustrate the frequencies of the change reasons over time.

**Results:**

Five hundred fifty-one patients were included of which 67.9% were female and oligoarticular JIA was the most common JIA diagnosis (41.9%). Thousand four hundred twenty-two different systemic drug treatment lines were observed, with a median number of 2 (IQR 1–3) treatment lines per patient. MTX was predominantly used as first treatment line (89.1%), while bDMARDs were mostly used as next treatment line (53.3%). JIA medication was mostly changed because of inefficacy (42.9%) in the first treatment line vs remission in the second treatment line (46.2%).

**Conclusion:**

JIA medication is mostly changed because of inefficacy in the first treatment line (predominantly MTX) vs remission in the second treatment line (roughly half on bDMARDs). This indicates that bDMARDs may be a more effective treatment than MTX in part of the JIA population, advocating reconsideration of current JIA treatment guidelines.

Rheumatology key messages

- Medication changes were frequent in children and adolescents with JIA with widely varying patterns.
- Inefficacy was mostly observed with first-line JIA treatment (mainly MTX), while remission was the predominant change reason for the second-line treatment (roughly 50% bDMARDs).
- This study highlights potential benefits of bDMARDs over MTX, encouraging reconsideration of current treatment guidelines.

## Introduction

JIA encompasses all forms of arthritis having an unknown aetiology, starting before the age of 16 and lasting at least 6 weeks [1]. It is one of the most prevalent chronic inflammatory diseases in childhood [2]. Pharmacological options have substantially expanded over the past decades from NSAIDs to conventional DMARDs (cDMARDs), such as MTX, and more recently the biological DMARDs (bDMARDs), such as TNFα-inhibitors [3]. Nevertheless, most JIA patients require at least one medication change at some point during their treatment [4–7].

Given the wide drug arsenal, debate is ongoing about the step-up approach and optimal treatment sequence in JIA patients (e.g. bDMARDs over cDMARDs as preferred first-line option) [8–11]. Detailed information on longitudinal medication pattern changes may provide insights on this topic. Overall, treatment adjustments are mostly because of remission, adverse events or lack of efficacy [6, 12], but current literature typically does not distinguish between individual treatment lines [7, 13]. Therefore, it is currently unknown whether these change reasons differ between treatment lines or whether recurring patterns exist (e.g. patients who continuously experience multi-drug-resistance or adverse reactions). Indeed, JIA patients who stopped TNFα-inhibitors due to adverse events were at higher risk of discontinuing later therapies for similar reasons [14], and this has been observed in other diseases as well [15–17]. This is important clinical information for shared decision making and to select individual treatment [18, 19]. Finally, zooming in on individual treatment lines allows real-world comparisons between specific treatment modalities, for example MTX (mostly given as first-line treatment) vs other medications.

The current study therefore aims to provide insight into longitudinal change patterns of systemic medication (systemic corticosteroids, cDMARDs and bDMARDs) using a real-world cohort of children and adolescents with JIA. We investigated (1) why the decision was made to change medication (*change reason*) during the whole treatment, (2) when the decision was made to change medication (*time to change*) and (3) which patterns of change reasons occur within a patient.

## Patients and methods

### Setting, study design and study population

We conducted a retrospective observational follow-up study with prospectively documented data from the Wilhelmina Children’s Hospital (WCH, part of the University Medical Center Utrecht in The Netherlands), a large tertiary referral centre for paediatric rheumatology. Each year ∼400 JIA patients visit this academic centre, of whom 65 are newly diagnosed. During these visits, disease-specific information is collected in the electronic patient file (EPF). In addition, since 2011, the paediatric rheumatologists use a structured way to collect the following medication data for JIA patients: start date, name of DMARD, stop date, stop reason and remarks. For patients already visiting a paediatric rheumatologist at the time of introduction of this method, data before 2011 were registered retrospectively based on information in the EPF, after which follow-up became prospective. Data were extracted pseudonymized to a previously developed research data platform [20], which was employed successfully before [6]. Using these data was classified by the institutional review board as exempt from the Medical Research Involving Human Subjects Act (11/499 and 18/474). This study was conducted according to Good Clinical Practice guidelines and the declaration of Helsinki [21].

Patients were eligible for the present study if they were aged <18 years at start of systemic medication, were diagnosed with JIA as defined by the International League of Associations for Rheumatology (ILAR) classification [1] and had received their first systemic treatment in the study centre between 1 September 1997 and 1 March 2024. Cohort entry was defined as the first systemic medication prescription (Supplementary Fig. S1). Systemic medication was defined as systemic corticosteroids (prednisolone, dexamethasone, hydrocortisone and methylprednisolone), cDMARDs (MTX, LEF, HCQ, mycophenolic acid, AZA and SSZ), bDMARDs (abatacept, adalimumab, certolizumab, etanercept, infliximab, golimumab, rituximab, sarilumab, tocilizumab and ustekinumab), tofacitinib and other anti-inflammatory drugs (colchicine, sirolimus). NSAIDs (e.g. diclofenac) were not part of this definition since their use is not restricted to JIA and can be used over the counter. Besides, systemic corticosteroids used as pulse therapy, defined by the treating paediatric rheumatologist, to treat flares were not included as systemic medication. Patients with systemic JIA were excluded since their treatment differs significantly from other JIA types [22]. Patients were also excluded if they already received systemic therapy >30 days before their first rheumatology-related appointment at the WCH as this may reflect medication use for another indication or visiting the WCH for a second opinion or tertiary referral (Supplementary Figs S1 and S2). Follow-up ended at the age of 18 years, the last rheumatology-related appointment or the end of study period (Supplementary Fig. S1).

### Definitions of treatment trajectories and treatment lines

**Figure 1:**
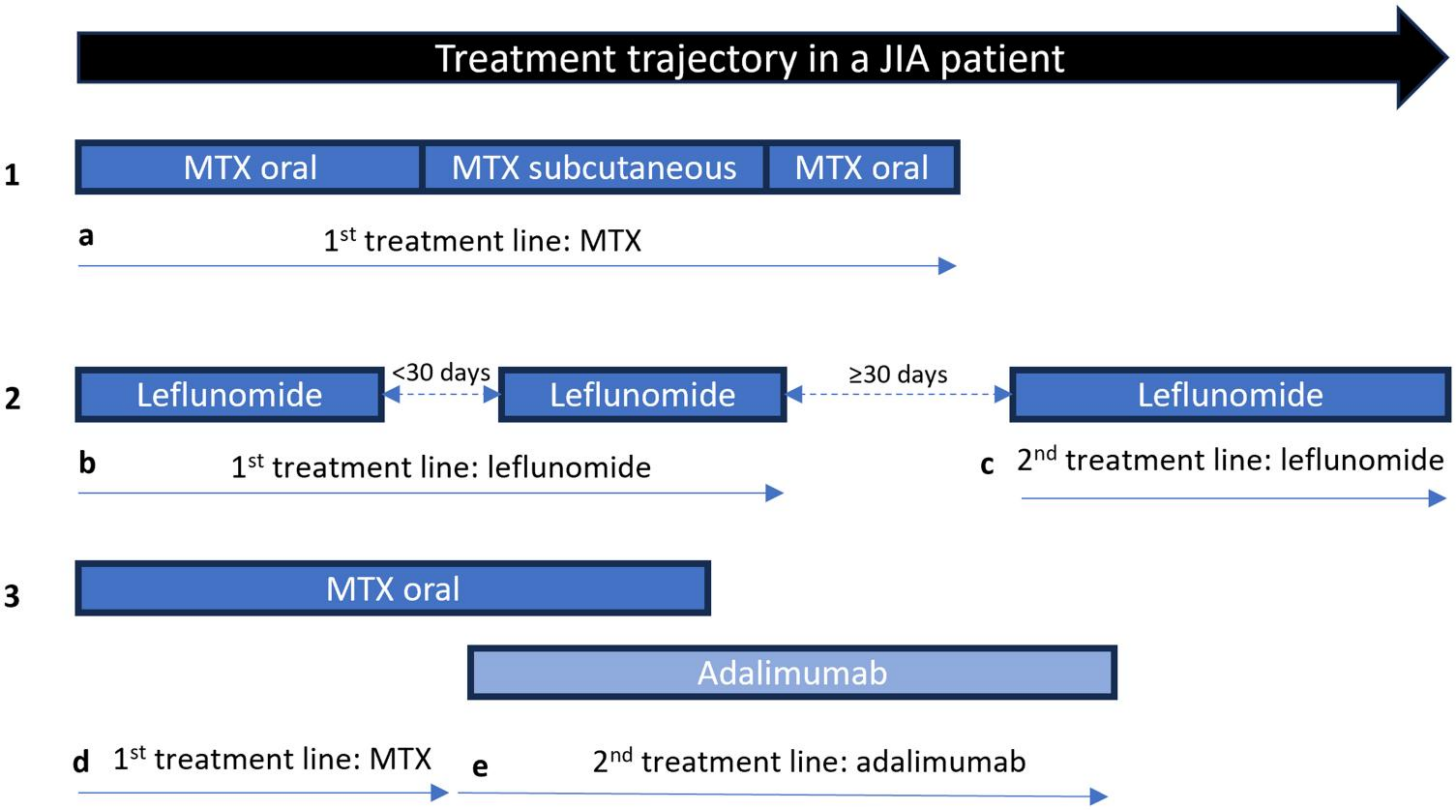
Hypothetical treatment trajectories of three JIA patients to visualize the definition of treatment lines. For each patient a treatment trajectory, containing the treatment lines, was created. Duration of the treatment lines was based on the start and stop dates entered in the electronic patient files, with the following exceptions: (**1**) switch between oral and subcutaneous MTX or *vice versa* if the change was within 1 month (**a**), (**2**) switch between tocilizumab intravenous and subcutaneous when occurred within 1 month and (**3**) restart of the same medication within 1 month (**b**). Dosage adjustments were not seen as a change. Treatment lines were based on the main systemic medication and not on JIA-related comedication. The use of systemic corticosteroids was classified as comedication in conjunction with cDMARDs and/or bDMARDs, and the use of a cDMARD next to a bDMARD was also classified as comedication. Directly at commencing a bDMARD, the treatment line including systemic corticosteroids and/or cDMARDs as main effective medication was ended (**d**). The discontinuation of comedication was not included in the analysis. So, a bDMARD combined with comedication followed by the same bDMARD without comedication was seen as one bDMARD treatment line (**e**)

We derived drug treatment trajectories for each patient describing the JIA medication journey over time (Fig. 1). Each trajectory started with the first prescription of systemic treatment and ended when the last systemic therapy stopped or follow-up ended, whichever came first. The trajectory included all treatment lines containing a systemic drug. A treatment line was defined as the period in which a (combination of) systemic drug(s) was used without a change in main treatment. Dosage adjustments were not seen as change. Duration of the different treatment lines was based on the start and stop dates entered in the EPF, excluding changes in administration routes or restarting the same medication within 1 month.

For the analysis, we focused on the main effective medication and not on JIA-related comedication. We used the following treatment hierarchy: bDMARDs above cDMARDs, and cDMARDs above corticosteroids. Directly at commencing a treatment higher in hierarchy, the other treatment was considered comedication. Discontinuation of comedication was not included in the analysis.

### Reasons for medication change

Our primary outcome was the reason for medication change. The treating paediatric rheumatologist prospectively registered the reason for any change in systemic medication. Reported reasons were mutually exclusive and included:

- Remission: the achievement of inactive disease, defined as an active joint count of zero and no uveitis;
- Adverse events: the occurrence of a harmful or unwanted event, including intolerance, that outweighs the potential benefits of the current medication based on both the patient’s and clinician’s perspective;
- Ineffectiveness: the inability to achieve inactive disease with the current medication or worsening of the disease despite the current medication;
- Other: all change reasons that did not belong to one of the before mentioned categories.

The label ‘no change’ was added in case a patient still used the systemic medication at the end of follow-up.

### Time to change

In addition, the time to reason-specific change was calculated. Time to change was defined as the time from start of systemic medication till the moment systemic medication was registered as changed in the EPF as described above. We did not study the drug free time between the stop and (re)start of medication.

### Covariables

Additional data on covariables were collected to describe the study population and stratification purposes. These included age at start of systemic medication, biological sex, date of first rheumatologic appointment and ILAR JIA category as reported by the physician in the EPF.

### Data analysis

A Sankey diagram visualized flows between different change reasons during a patient’s treatment trajectory. Cumulative incidences curves were used to show frequencies of the change reasons over time, taking competing risks into consideration (Fine-Gray models). Data were stratified by number of treatment line and separate plots per change reason were made to compare them. Wilcoxon rank tests were performed to compare the time to change per reason between the treatment lines. In addition, we included ‘previous change reason’ to examine whether patients experienced the same change reason consecutively. We also checked for a calendar time effect. The covariables age, biological sex, primary date of diagnosis and JIA subtype were only used to describe the study population.

Because the risk of missing data was theoretically present, a missing data analysis was done by comparing the use of bDMARDs in the structurally collected data in the EPF to the dispensing information of the outpatient pharmacy. In the Netherlands, all bDMARDs are exclusively dispensed by the outpatient pharmacy of the hospital where a patient is treated due to reimbursement regulations. Consequently, the outpatient pharmacy contains a complete overview of all biological drugs used in the home setting.

All analyses were performed with R (version 4.4.0, Posit Software, PBC, Boston, Massachusetts) and the packages ggplot2, tidycmprsk, survival and networkD3 [23–26].

## Results

A total of 551 patients met the inclusion criteria (Table 1 and Supplementary Fig. S2), using 1422 different drug treatment lines together during the study period. Median follow-up per patient was 5.7 years (interquartile range (IQR) 3.0–10.6 years) and the median number of treatment lines was 2 (IQR 1–3) per patient. The median age at start of systemic medication was 9.5 years (IQR 4.2–13.8 years) and about two-thirds of the patients was female (67.9%). The most common diagnosis was oligoarticular JIA (41.9%), and the distribution of JIA subtypes was similar for the different treatment lines. The time between the first rheumatologic appointment and the start of systemic medication was longer for patients starting with systemic medication between 1997 and 2010 compared with the patients starting between 2011 and 2017 or between 2018 and 2024 (2.2 months (IQR 0.4–10.4) vs 1.4 months (0.0–8.2, *P* = 0.23) and 1.2 months (0.0–5.0, *P* = 0.015)).

**Table 1:** Patient characteristics.

	Overall (*n* = 551)
Follow-up since start systemic medication in years, median (IQR)	5.7 [3.0–10.6]
Number of treatment lines, median (IQR)	2.0 [1.0–3.0]
Age at start systemic medication in years, median (IQR)	9.5 [4.2–13.8]
Biological sex (female), % (*n*)	67.9% (374)
ILAR category of JIA, *n* (%)	
Oligoarticular JIA	41.9% (231)
Enthesitis-related JIA	5.1% (28)
Polyarticular RF-negative JIA	23.4% (129)
Polyarticular RF-positive JIA	6.9% (38)
Psoriatic JIA	4.0% (22)
Undifferentiated JIA	14.7% (81)
Unknown	4.0% (22)
Time between first rheumatologic appointment and start first systemic medication per start period in months, median (IQR)	
1997–2010 (*n* = 139)	2.2 (0.4–10.4)
2011–2017 (*n* = 246)	1.4 (0.0–8.2)
2018–2024 (*n* = 166)	1.1 (0.0–5.0)

IQR, interquartile range.

MTX was the most frequently used medication in treatment line 1 (89.1%, *n* = 491), with diminishing use in further treatment lines (Table 2). In contrast, in treatment line 2 half of the patients used a TNFα-inhibitor (52.0%, *n* = 197). Over the next treatment lines, the use of other bDMARDs increased (from 1.3% in treatment line 2 to 15.1% in treatment line 5), while the use of TNFα-inhibitors remained stable (45–55%). Adalimumab was the most frequently used TNFα-inhibitor (34.0% in treatment line 2), followed by etanercept (16.4% in treatment line 2). Despite the fact that the use of TNFα-inhibitors as first treatment line is only on-label for enthesitis-related JIA, we observed that these were also used for few individuals with other JIA subtypes in treatment line 1 (data not shown). Good concordance was observed between the medication data structurally collected via the EPF and the dispensing data recorded by the outpatient pharmacy.

**Table 2:** Overview of medication used in treatment lines 1–5, including follow-up time and change reason.

Treatment line	1 (*N* = 551)	2 (*N* = 379)	3 (*N* = 222)	4 (*N* = 134)	5 (*N* = 73)
cDMARDs, % (*n*)	92.7% (511)	44.9% (170)	45.0% (100)	35.1% (47)	26.0% (19)
Prescribed cDMARDs, % (*n*)					
MTX	89.1% (491)	34.0% (129)	33.3% (74)	23.9% (32)	17.8% (13)
LEF	0.5% (3)	7.1% (27)	8.1% (18)	9.0% (12)	5.5% (4)
Other cDMARD	3.1% (17)	2.1% (8)	3.2% (7)	1.5% (2)	1.4% (1)
Combined cDMARD	0% (0)	1,8% (7)	0.5% (1)	0.7% (1)	1.4% (1)
TNFα-inhibitors, % (*n*)	6.5% (36)	52.0% (197)	46.4% (103)	51.5% (69)	54.8% (40)
Prescribed TNFα-inhibitors, % (*n*)					
Adalimumab	3.1% (17)	34.0% (129)	27.5% (61)	22.4% (30)	19.2% (14)
Etanercept	2.5% (14)	16.4% (62)	15.8% (35)	20.1% (27)	17.8% (13)
Golimumab	0.9% (5)	1.3% (5)	2.7% (6)	4.5% (6)	15.1% (11)
Infliximab	0% (0)	0.3% (1)	0.5% (1)	4.5% (6)	2.7% (2)
Other bDMARDs, % (*n*)	0% (0)	1.3% (5)	6.3% (14)	11.2% (15)	15.1% (11)
Prescribed other bDMARDs, % (n)					
Abatacept	0% (0)	0% (0)	0.5% (1)	3.7% (5)	2.7% (2)
Tocilizumab	0% (0)	1.3% (5)	5.9% (13)	7.5% (10)	12.3% (9)
Other main treatments, % (*n*)	0.7% (4)	1.8% (7)	2.3% (5)	2.2% (3)	4.1% (3)
Prescribed other main treatments, % (*n*)					
Systemic corticosteroid	0.7% (4)	0.5% (2)	0.5% (1)	0.7% (1)	0% (0)
Tofacitinib	0% (0)	1.1% (4)	1.8% (4)	1.5% (2)	4.1% (3)
Other anti-inflammatory drug	0% (0)	0.3% (1)	0% (0)	0% (0)	0% (0)
Time to change or to end of follow-up in months, median (IQR)	12.9 [5.2–24.8]	16.8 [8.0–28.1]	12.9 [6.2–25.5]	14.5 [5.0–30.1]	11.5 [3.9–25.5]
Change, % of total (*n*)	85.5% (471)	72.0% (273)	70.3% (156)	61.2% (82)	49.3% (36)
By change reason, expressed as % of changers (*n*)					
Remission	36.3% (171)	46.2% (126)	39.1% (61)	25.6% (21)	22.2% (8)
Inefficacy	42.9% (202)	34.1% (93)	36.5% (57)	51.2% (42)	58.3% (21)
Adverse event	18.7% (88)	14.7% (40)	20.5% (32)	17.1% (14)	16.7% (6)
Other	2.1% (10)	5.1% (14)	3.8% (6)	6.1% (5)	2.8% (1)
No change, % of total (*n*)	14.5% (80)	28.0% (106)	29.7% (66)	38.8% (52)	50.7% (37)

This table shows per treatment line the number and percentage of JIA patients who had cDMARDs, TNFα-inhibitors, other bDMARDs and other medicines as main effective drug. The median follow-up and the percentage of the change reason are also shown per treatment line.

bDMARD, biological DMARD; cDMARD, conventional DMARD; IQR, interquartile range.

Treatment lines 1–5 are shown in the table. Data of the other treatment lines can be found in Table S1 as <10% of the patients reaches treatment line 6 or more within the observation period.

**Figure 2:**
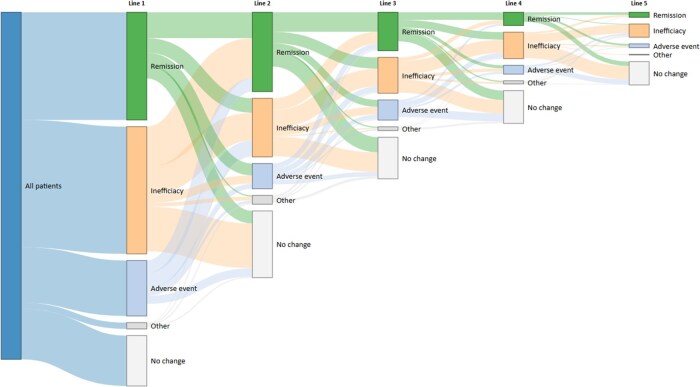
Sankey diagram showing the sequences of change reasons for systemic medication. This figure visualizes the order of reasons to change systemic medication in the treatment lines 1–5, regardless of treatment duration. No change means that a patient still used the medication at end of follow-up. The width of each flow is proportional to the number of patients. The higher inflow relative to the outflow reflects that not all patients transitioned to a next treatment line after stopping. Follow-up of patients was censored at the age of 18 years, the last rheumatology-related appointment or the end of study period. The Sankey diagram shows that overall 40% of the patients moves on to next treatment line each time, and that almost all patients initiated another treatment line when they stopped the previous line because of inefficacy (orange) or adverse event (blue). This was consistent over the treatment lines. Patients who experienced remission (green) started a subsequent treatment line less frequently as shown by the smaller outflows compared with the inflows. Treatment lines 1–5 are shown in the Sankey diagram. Data of the other treatment lines is not shown as less than 10% of the patients reaches treatment line 6 or more within the observation period

Of the 551 patients, 379 (68.8%) continued with a second treatment line (Table 2). This percentage decreased with each treatment line; 40.3% of all patients needed treatment line 3, 24.3% treatment line 4 and 13.2% treatment line 5. This trend was also evident from the Sankey diagram (Fig. 2). Median time % to event or end of follow-up was approximately 1 year in treatment lines 1, 3 and 5, whereas it was longer in treatment lines 2 and 4 (respectively 16.8 and 14.5 months).

### Reasons for medication change

**Figure 3:**
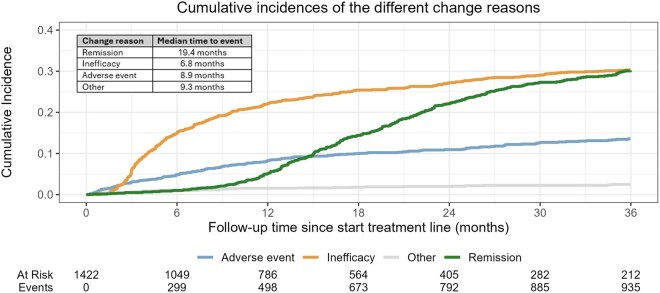
Cumulative incidence curves of the different change reasons. This figure presents the overall cumulative incidence of the reasons for changing systemic medication, regardless of the treatment line. The median time to event per change reason and the numbers at risk are also shown. Inefficacy was overall the most observed change reason for changing systemic medication, followed by remission. Adverse event was the least observed main change reason. The median time to change systemic treatment differed between the change reasons. Systemic medication was used for the longest period when patients eventually changed because of remission, and the shortest period when patients eventually changed because of inefficacy

Overall, inefficacy was the most observed change reason, followed by remission. Adverse event was the least observed main change reason (Fig. 3). As expected on clinical experience, nausea and vomiting were the most reported adverse events in treatment line 1. In addition, elevation of liver enzymes was mentioned several times, and two patients developed a serious infection while using MTX. Reasons classified as ‘other’ were diverse, such as not wanting to take medication anymore due to the COVID pandemic, adherence issues or fear of long-term side effects. The change reasons per individual treatment line are visualized in Fig. 2 and percentages are displayed in Table 2. The distribution of change reasons is comparable for the different start periods (Supplementary Fig. S3), except for ‘no change’ which is higher in the most recent period and ‘remission’ which is lower in the most recent period. The distribution is also comparable for almost all JIA subtypes in all treatment lines (Supplementary Fig. S4A), in treatment line 1 (Supplementary Fig. S4B) and for MTX in treatment line 1 (data not shown). In oligoarticular JIA, however, more remission was seen compared with for example enthesitis-related JIA and undifferentiated JIA (33.4% vs 20.7% and 21.2%).

The Sankey diagram displays a substantial difference in changes reasons between treatment lines 1 and 2. Inefficacy (42.9%) was the most prevalent reason in treatment line 1, followed by remission (36.3%) and adverse event (18.7%). In contrast, during treatment line 2, remission (46.2%) was the most common, rather than inefficacy (34.1%). Treatment line 3 followed similar patterns as line 2, while in treatment lines 4 and 5, the flows towards inefficacy were again the widest; the contribution of this change reason was even higher than in treatment line 1 (respectively 51.2% and 58.3% vs 42.9%).

**Figure 4:**
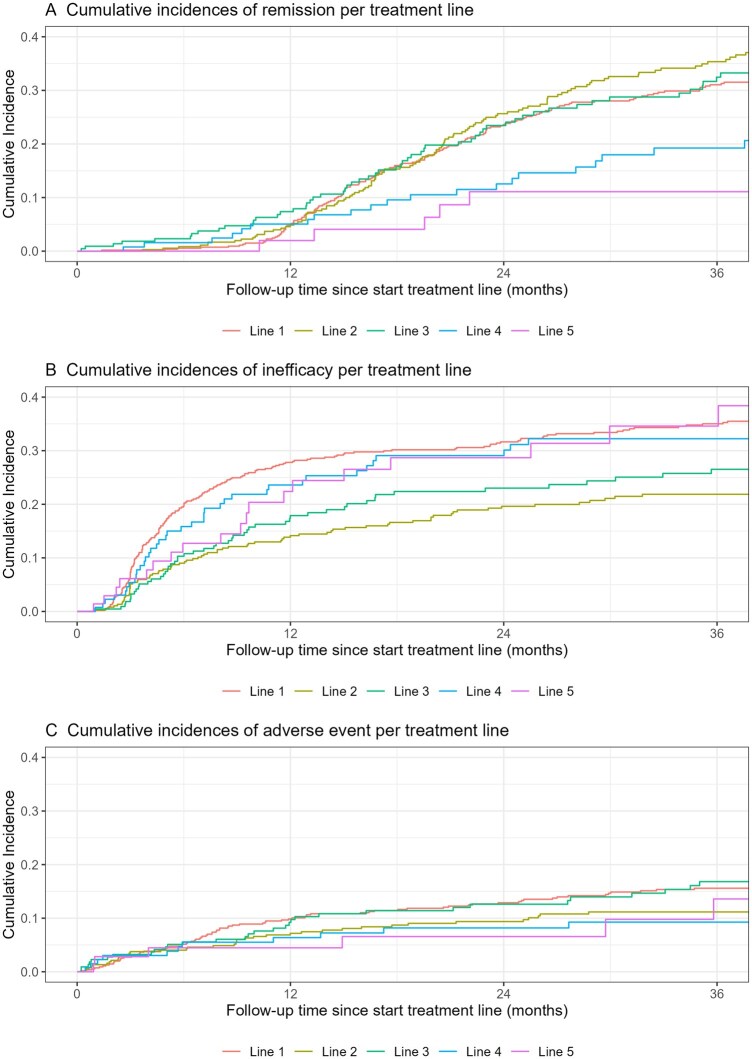
Cumulative incidences curves for the different change reasons per treatment line. Panel **A** shows the incidences for remission, Panel **B** for inefficacy and Panel **C** for adverse event. For remission, the incidence was substantially lower in treatment lines 4 and 5, whereas the incidence of remission was higher in treatment line 2 compared with treatment lines 1 and 3 (Panel **A**). For inefficacy, the incidence was considerably lower in treatment line 2 compared with the others (Panel **B**). The highest incidence of inefficacy was observed in treatment line 5, while treatment line 1 also showed a relatively high incidence of inefficacy compared with treatment line 3 and 4. For adverse events, the incidence did not differ between the treatment lines (Panel **C**). Treatment lines 1–5 are shown in the figures. Data of the other treatment lines are not shown as less than 10% of the patients reaches treatment line 6 or more within the observation period

To further investigate whether the incidence of the change reasons was affected by the number of treatment lines, data were plotted for individual treatment lines in Fig. 4. For adverse events, the incidence did not differ between the treatment lines (Panel A). For inefficacy, the incidence was considerably lower in treatment line 2 compared with the others (Panel B). The highest incidence of inefficacy was observed in treatment line 5, while treatment line 1 also showed a relatively high incidence of inefficacy compared with treatment lines 3 and 4. For remission, the incidence was substantially lower in treatment lines 4 and 5, whereas the incidence of remission was higher in treatment line 2 compared with treatment lines 1 and 3 (Panel C).

### Time to change

The median time to change systemic treatment differed between the change reasons. Systemic medication was used the longest when patients eventually changed because of remission; 19.4 months of median use (IQR 13.4–26.5) compared with 6.8 (3.4–16.8) and 8.9 (3.5–21.0) months for respectively inefficacy and adverse event. Zooming in again per treatment line revealed no significant differences for the change reasons ‘remission’ and ‘adverse event’. However, for ‘inefficacy’, the median time to event in treatment line 2 was significantly longer compared with treatment lines 1, 3 and 5 (10.0 (4.1–28.3) months compared with for example 5.5 (3.2–12.2) months in treatment line 1; data can be found in Supplementary Table S2).

### Patterns of medication changes

The Sankey diagram (Fig. 2) shows that almost all patients initiated another treatment line when they stopped the previous line because of inefficacy or adverse event. This was consistent over the treatment lines. Patients who experienced remission started a subsequent treatment line less frequently as shown by the smaller outflow compared with the inflow. However, still about 60% of the patients did start treatment line 2 despite experiencing remission in treatment line 1. This percentage increased over the treatment lines (outflow became more comparable to inflow), for example almost 75% in treatment line 4, showing that in further treatment lines more patients started a subsequent treatment line despite having remission before. Another observation made from the Sankey diagram was that half of the patients who achieved remission with treatment line 2 had previously experienced inefficacy. It was also apparent that, from treatment line 2 onwards, a larger proportion of patients did not change their JIA medication compared with treatment line 1 (percentages shown in Table 2).

To investigate whether a pattern existed in the consecutive change reasons, we explored how often the same change reason was reported multiple times in a row. The percentage of patients with two consecutive changes, restricted to the ascribed treatment line and the one before, because of inefficacy increased from treatment line 3 onwards (49.1%, 50.0% and 66.7% in, respectively, treatment lines 3, 4 and 5). Fifteen of the 57 (26.3%) patients reporting inefficacy in treatment line 3 experienced inefficacy again in both treatment lines 4 and 5. This proportion was considerably higher than expected based on a random and even distribution of change reasons (7.8%) and included 2.7% of the total population. Only a small proportion of the patients (1.5%, eight out of 551) had had consecutive inefficacy from treatment lines 1 up to 4 (for data see Supplementary Table S3); based on a random and even distribution of change reason, 1.2% was expected. For adverse events, no pattern was observed as only 1.5% of the patients experienced adverse events again in treatment line 2 and 0.2% experienced adverse events for a third time in treatment line 3.

## Discussion

This article describes the longitudinal change patterns of systemic medication in a large real-world cohort of children and adolescents with JIA. The results show a marked difference in change patterns between lines 1 (mainly MTX) and line 2 (50% bDMARDs). Inefficacy (42.9%) was the main reason of medication change in treatment line 1, whereas in treatment line 2 most patients discontinued medication because of remission (46.2%). The incidence of adverse events was comparable across different treatment lines and was relatively low in all of them, indicating that JIA medications were well tolerated by the majority of patients and that most changes were related to disease activity and not medication safety. We observed a significantly longer duration to medication change for inefficacy in treatment line 2 compared with treatment line 1. The duration to adverse event and remission were similar for all treatment lines. According to the patterns in change reasons, we observed that a large percentage of the patients did start a subsequent treatment line despite having remission before, most likely this reflects the known inability to stop the medication for a longer period in >70% of the JIA patients [27, 28]. It was also seen that it was difficult to try to change again because of remission after experiencing inefficacy in treatment line 3.

The observed difference in main change reason between treatment lines 1 and 2, inefficacy vs remission, may be explained by the large differences in medication used in these treatment lines, and potentially calls for guideline reconsiderations. In line with current guideline recommendations, in which a step-up approach with first cDMARDs and thereafter bDMARDs is recommended [22], MTX was the most frequently used drug in treatment line 1 and TNFα-inhibitors in treatment line 2. Previous real-world data studies showed similar prescription patterns [6, 12]. However, the current step-up approach is partly based on the historic high costs of original bDMARDs [8, 9, 22], resulting in a lack of cost-effectiveness for bDMARDs. Less expensive biosimilars are currently available, and Tarkiainen *et al.* [10] showed that using biosimilar infliximab plus MTX as a first-line treatment can be considered cost-effective compared with MTX alone. Therefore, reconsideration of the current treatment order might be warranted. This is also supported by the study of de Jonge *et al.* [11], in which they showed that better disease control was reached when bDMARDs were initiated early after symptom onset instead of later in the disease course. The authors suggest the existence of a window of opportunity for the treatment with bDMARDs, which we might not optimally utilize since we first start with MTX. Our observations are in line with this and raise the question whether the current sequence in therapies is optimal for the majority of JIA patients. For a proportion of them, MTX remains an appropriate first-line treatment option to achieve disease control, but there seems to be a large group of patients who would benefit more from earlier initiation of bDMARDs. Patient characteristics that can help distinguish between patients who benefit from MTX as a first-line treatment and those who do not should be identified in future research.

The time to change due to inefficacy was longer in treatment line 2 compared with treatment line 1. The development of anti-drug antibodies (ADAs) against bDMARDs, the most frequently used drugs in treatment line 2, must be considered. However, data about the formation of ADAs were not available as ADAs were not structurally measured in the WCH when patients experienced inefficacy. Formation of ADAs can occur later during treatment, resulting in loss of efficacy. Since loss of efficacy was included in the change reason ‘inefficacy’ in this study, this might have resulted in a longer time to change for inefficacy in treatment line 2 compared with treatment line 1 in which almost no bDMARDs were used. As bDMARDs were also frequently used in treatment line 3 onwards, we would expect a longer time to change due to inefficacy in those treatment lines as well. Although not significantly different compared with treatment line 1, a trend to a longer time to change due to inefficacy was indeed observed in treatment line 3 onwards. Other explanations can also be taken into account. For example, patients starting with treatment line 2 might had a more severe disease activity due to ineffectiveness of treatment line 1, resulting in early partial responses with treatment line 2 and that clinicians wait longer to determine whether the medication achieves complete disease control. This is in contrast with the treat-to-target strategy commonly used in JIA patients since 2019, which aims to evaluate treatment effectiveness after 6 months and adjust therapy if complete disease control is not yet achieved [29].

When exploring the possible sequential patterns of change reasons, we observed that a large proportion of the patients experienced repeated inefficacy consecutively in treatment lines 3–5. Therefore, experiencing inefficacy in treatment line 3 appears to be associated with a poor prognosis for achieving disease control, suggesting the presence of a subpopulation with a treatment-resistant JIA form. This subpopulation was not limited to a specific JIA subtype as the distribution of JIA subtypes between patients who required a fourth treatment line was comparable to that of the overall cohort. The size of the subpopulation with a treatment-resistant JIA form may actually be underestimated based on this study as follow-up was limited by the age of 18 years or the end of the study period.

Our results suggest that future research should focus on those patients whose treatment can be safely discontinued after reaching remission. Most patients initiated a new treatment line after a change—not only in those with adverse events or inefficacy, but ultimately in those in remission as well, indicating a disease flare. We did not include the drug free time between treatment lines, but studying this could provide valuable insights into how quickly flares occur and whether treatment discontinuation is truly beneficial and justified. Examining the drug free time can help distinguish between patients who really benefit from stopping because of remission and those who do not. In this context, it would also be interesting to investigate whether differences exist in achieving remission on medication again between patients who used the same drug again (restart), a drug with a similar mechanism of action (cycle) or a drug with another mechanism of action (switch).

This study has several strengths. First, data were collected prospectively and systematically recorded, which minimizes recall bias. Second, data reliability was high as cross-checking with outpatient pharmacy records revealed no significant discrepancies. Finally, the relatively large cohort allowed for a detailed examination of longitudinal treatment patterns in JIA patients.

Several limitations should also be acknowledged. First, the study was conducted at a single tertiary centre, which may limit generalizability. However, we classify our cohort as representative for the European JIA population as initiation of biologic therapy predominantly occurs in tertiary centres in this part of the world. However, it should be mentioned that JIA population differs between continents [30]. Second, selection bias may have occurred, as participation depended on physician documentation of treatment changes. Third, misclassification was possible, for example, when both adverse events and inefficacy occurred at the same time, but just one was recorded as the main change reason. In addition, misattribution of the change reason could have occurred in the presence of co-mediation. Finally, calendar time effects may have influenced treatment practices during the study period, potentially affecting treatment duration and switching patterns. For example, Lamers *et al.* [31] showed the increased use of bDMARDs between 2012 and 2024 and the switch from etanercept to adalimumab as most used TNFα-inhibitor after change in adalimumab formulation. In our study, we checked the distribution of the change reasons in three starting periods and the distribution was comparable between those periods, except for ‘no change’ and ‘remission’ in the last period. This can be explained by the fact that the patients in the last period were more recently included and had therefore less time to already change their medication, and remission is a typical late onset change reason in contrast to adverse event and inefficacy. Despite the mentioned limitations, we consider our findings to be representative in illustrating the frequency, timing and patterns of change reasons for systemic medication in JIA patients.

In conclusion, first-line JIA treatment (mainly MTX) is mostly changed because of inefficacy (42.9%), whereas remission was the dominant changing reason in the second treatment line (roughly half on bDMARDs) in children and adolescents. This indicates that bDMARDs may be a more effective treatment than MTX in at least a large part of the JIA population, advocating reconsideration of current JIA-treatment guidelines.

## Supplementary Material

keag296_Supplementary_Data

## Data Availability

The data that support the findings of this study are available from the corresponding author, upon reasonable request.
