# Supplementary material for: Changes in systemic medication in children and adolescents with JIA: why, when and which patterns
Source: Rheumatology (Oxford). 2026 Jun 10;65(6):keag296. doi: 10.1093/rheumatology/keag296 (PMC13312428; doi:10.1093/rheumatology/keag296)
Supplement: keag296_Supplementary_Data [file keag296_supplementary_data.docx]

## Supplementary material


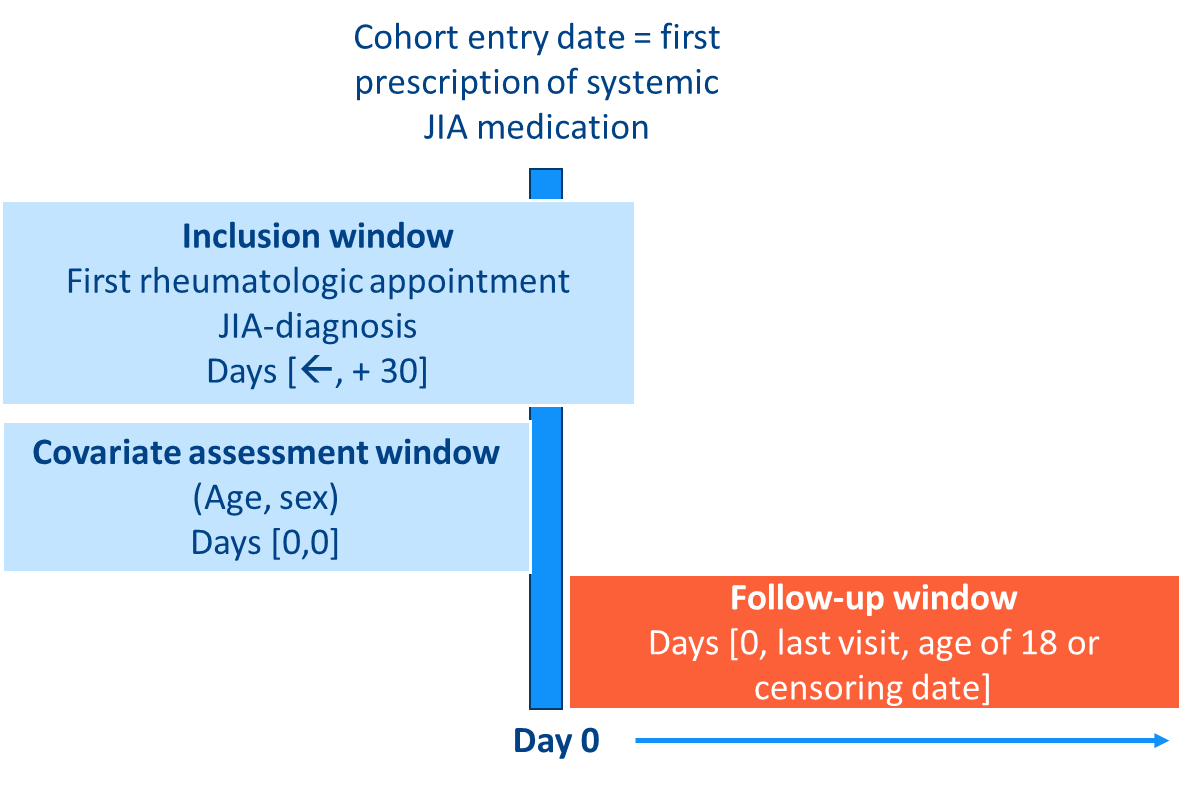


**Figure S1.** **Graphical depiction of study design.** Patients entered the cohort at the moment of first prescription of systemic JIA medication. Data about covariates were collected retrospectively, while medication data were collected prospectively during follow-up. Follow-up ended at the age of 18 years, the last rheumatology-related appointment or the end of study period.


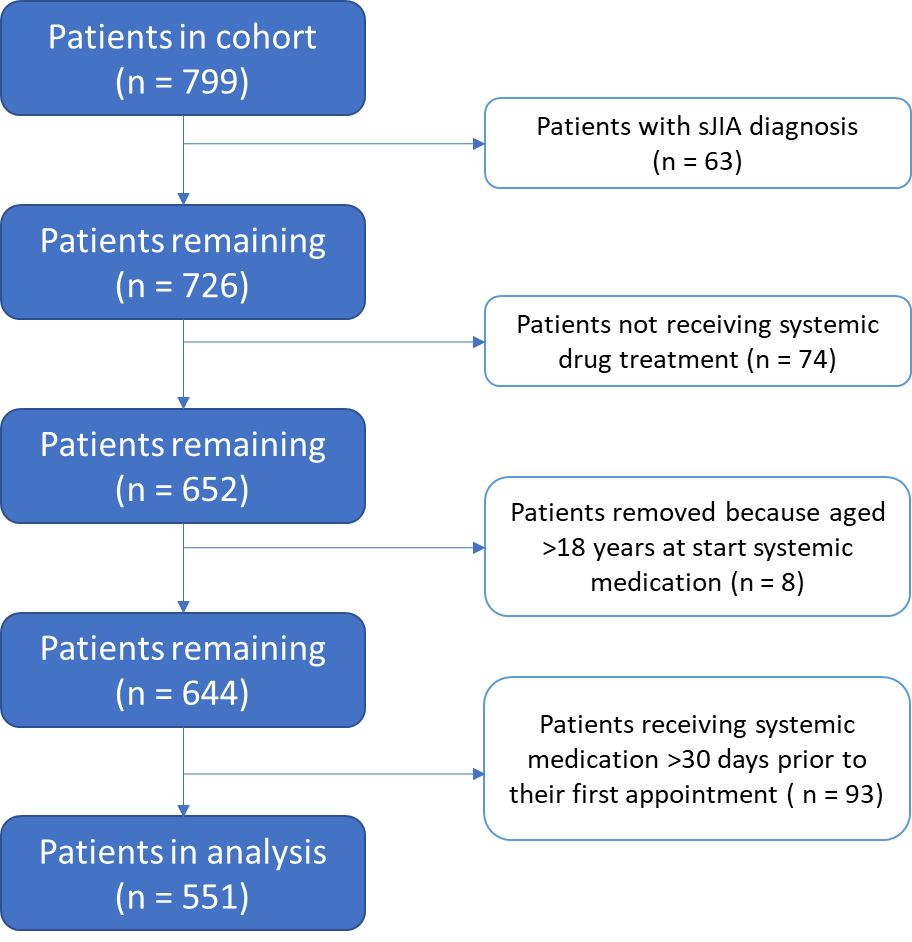


**Figure S2.** Overview of patient exclusion from the cohort.


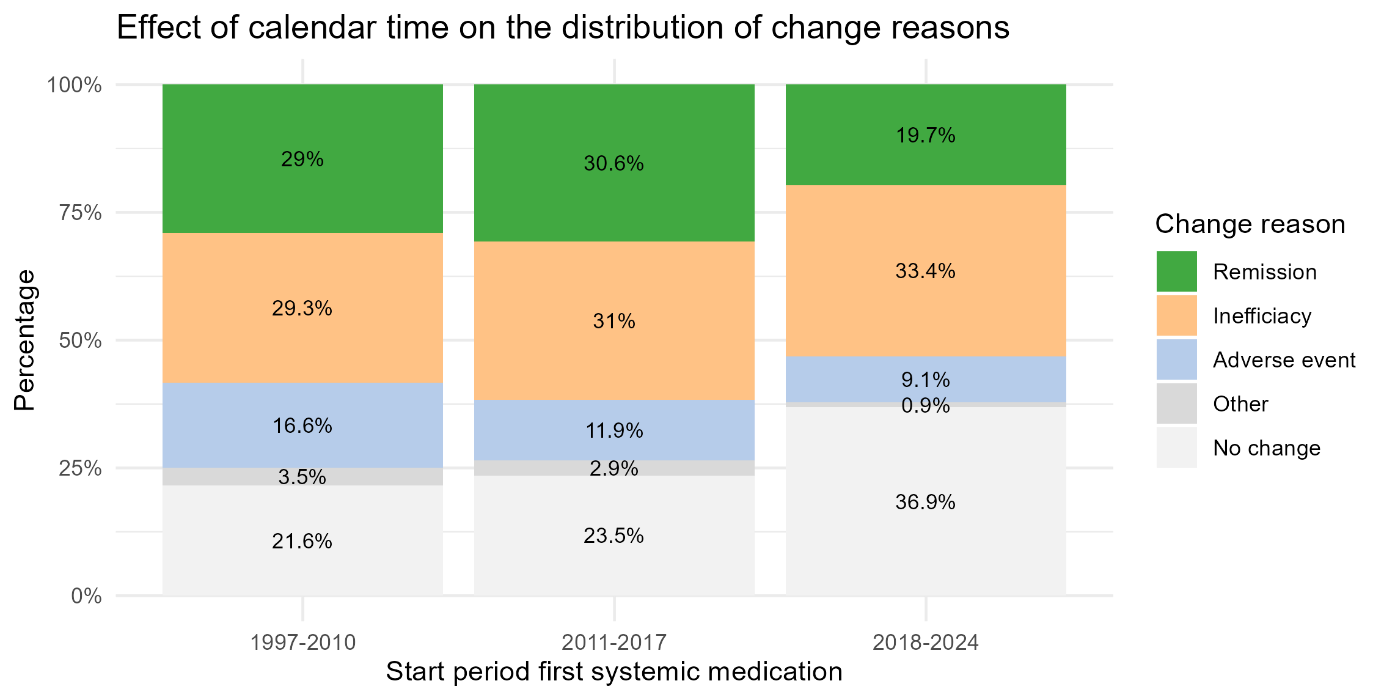


***Figure S3. Effect of calendar time on the distribution of change reasons****.*

*
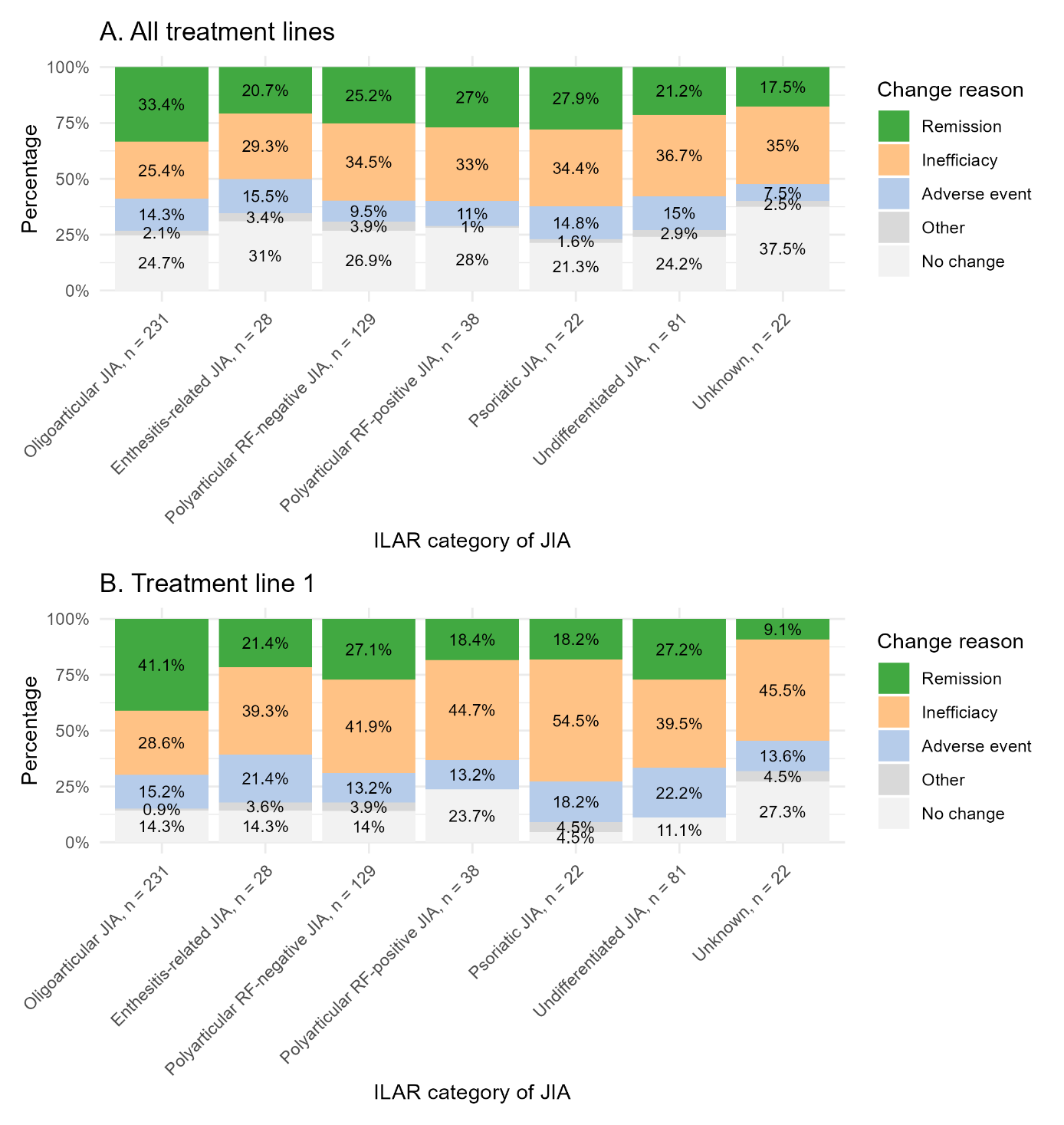
****Figure S4. Distribution of change reasons per ILAR category of JIA.*** *A) All treatment lines are included in the figure. B) Only treatment line 1 (mainly MTX) is shown in the figure.*

**Table S1. Overview of medication used over the different treatment lines, including follow-up time and change reason.** This table shows per treatment line the number and percentage of JIA patients who had cDMARDs, TNFα-inhibitors, other bDMARDs and other bDMARDs and other medicines as main effective drug. The median follow-up and the percentage of the change reason are also shown per treatment line.

| **Treatment line** | **1 (N=551)** | **2 (N=379)** | **3 (N=222)** | **4 (N=134)** | **5 (N=73)** | **6 (N=31)** | **7 (N=12)** | **8 (N=8)** | **9 (N=6)** | **10 (N=5)** | **11 (N=1)** | **Overall (N=1422)** |
| --- | --- | --- | --- | --- | --- | --- | --- | --- | --- | --- | --- | --- |
| **cDMARDs, n (%)** | **92.7% (511)** | **44.9% (170)** | **45.0% (100)** | **35.1% (47)** | **26.0% (19)** | **29.0% (9)** | **25.0% (3)** | **12.5% (1)** | **0% (0)** | **0% (0)** | **0% (0)** | **60.5% (860)** |
| **Prescribed cDMARDs, n (%)** |  |  |  |  |  |  |  |  |  |  |  |  |
| MTX | 89.1% (491) | 34.0% (129) | 33.3% (74) | 23.9% (32) | 17.8% (13) | 12.9% (4) | 25.0% (3) | 0% (0) | 0% (0) | 0% (0) | 0% (0) | 52.5% (746) |
| LEF | 0.5% (3) | 7.1% (27) | 8.1% (18) | 9.0% (12) | 5.5% (4) | 12.9% (4) | 0% (0) | 12.5% (1) | 0% (0) | 0% (0) | 0% (0) | 4.9% (69) |
| Other cDMARD | 3.1% (17) | 2.1% (8) | 3.2% (7) | 1.5% (2) | 1.4% (1) | 0% (0) | 0% (0) | 0% (0) | 16.7% (1) | 0% (0) | 0% (0) | 2.5% (36) |
| Combined cDMARDs | 0% (0) | 1,8% (7) | 0.5% (1) | 0.7% (1) | 1.4% (1) | 1.3% (1) | 0% (0) | 0% (0) | 0% (0) | 0% (0) | 0% (0) | 0,8% (11) |
| **TNFα-inhibitors, n (%)** | **6.5% (36)** | **52.0% (197)** | **46.4% (103)** | **51.5% (69)** | **54.8% (40)** | **29.0% (9)** | **50.0% (6)** | **50.0% (4)** | **33.3% (2)** | **40.0% (2)** | **100% (1)** | **33.0% (469)** |
| **Prescribed TNFα-inhibitors, n (%)** |  |  |  |  |  |  |  |  |  |  |  |  |
| Adalimumab | 3.1% (17) | 34.0% (129) | 27.5% (61) | 22.4% (30) | 19.2% (14) | 19.4% (6) | 16.7% (2) | 12.5% (1) | 33.3% (2) | 0% (0) | 0% (0) | 18.4% (262) |
| Etanercept | 2.5% (14) | 16.4% (62) | 15.8% (35) | 20.1% (27) | 17.8% (13) | 6.5% (2) | 16.7% (2) | 12.5% (1) | 0% (0) | 40.0% (2) | 100% (1) | 11.2% (159) |
| Golimumab | 0.9% (5) | 1.3% (5) | 2.7% (6) | 4.5% (6) | 15.1% (11) | 3.2% (1) | 8.3% (1) | 25.0% (2) | 0% (0) | 0% (0) | 0% (0) | 2.6% (37) |
| Infliximab | 0% (0) | 0.3% (1) | 0.5% (1) | 4.5% (6) | 2.7% (2) | 0% (0) | 8.3% (1) | 0% (0) | 0% (0) | 0% (0) | 0% (0) | 0.8% (11) |
| **Other bDMARDs, n (%)** | **0% (0)** | **1.3% (5)** | **6.3% (14)** | **11.2% (15)** | **15.1% (11)** | **38.7% (12)** | **8.3% (1)** | **25.0% (2)** | **50.0% (3)** | **60.0% (3)** | **0% (0)** | **4.6% (66)** |
| **Prescribed other bDMARDs, n (%)** |  |  |  |  |  |  |  |  |  |  |  |  |
| Tocilizumab | 0% (0) | 1.3% (5) | 5.9% (13) | 7.5% (10) | 12.3% (9) | 19.4% (6) | 0% (0) | 25.0% (2) | 33.3% (2) | 40.0% (2) | 0% (0) | 3.4% (49) |
| Abatacept | 0% (0) | 0% (0) | 0.5% (1) | 3.7% (5) | 2.7% (2) | 12.9% (4) | 8.3% (1) | 0% (0) | 0% (0) | 20.0% (1) | 0% (0) | 1.0% (14) |
| Sarilumab | 0% (0) | 0% (0) | 0% (0) | 0% (0) | 0% (0) | 3.2% (1) | 0% (0) | 0% (0) | 0% (0) | 0% (0) | 0% (0) | 0.1% (1) |
| Ustekinumab | 0% (0) | 0% (0) | 0% (0) | 0% (0) | 0% (0) | 3.2% (1) | 0% (0) | 0% (0) | 16.7% (1) | 0% (0) | 0% (0) | 0.1% (2) |
| **Other main treatments, n (%)** | **0.7% (4)** | **1.8% (7)** | **2.3% (5)** | **2.2% (3)** | **4.1% (3)** | **3.2% (1)** | **16.7% (2)** | **12.5% (1)** | **16.7% (1)** | **0% (0)** | **0% (0)** | **1.9% (27)** |
| **Prescribed other main treatments** |  |  |  |  |  |  |  |  |  |  |  |  |
| Systemic corticosteroid | 0.7% (4) | 0.5% (2) | 0.5% (1) | 0.7% (1) | 0% (0) | 0% (0) | 8.3% (1) | 12.5% (1) | 0% (0) | 0% (0) | 0% (0) | 0.7% (10) |
| Other anti-inflammatory drug | 0% (0) | 0.3% (1) | 0% (0) | 0% (0) | 0% (0) | 0% (0) | 0% (0) | 0% (0) | 0% (0) | 0% (0) | 0% (0) | 0.1% (1) |
| Tofacitinib | 0% (0) | 1.1% (4) | 1.8% (4) | 1.5% (2) | 4.1% (3) | 3.2% (1) | 8.3% (1) | 0% (0) | 0% (0) | 0% (0) | 0% (0) | 1.1% (15) |
| Combination of cDMARD and other anti-inflammatory drug | 0% (0) | 0% (0) | 0% (0) | 0% (0) | 0% (0) | 0% (0) | 0% (0) | 0% (0) | 16.7% (1) | 0% (0) | 0% (0) | 0.1% (1) |
| **Time to event or to end of follow-up in months, median (IQR)** | **12.9 [5.2 - 24.8]** | **16.8 [8.0 - 28.1]** | **12.9 [6.2 - 25.5]** | **14.5 [5.0 - 30.1]** | **11.5 [3.9 - 25.5]** | **9.5 [3.2 - 19.4]** | **7.2 [4.1 - 32.8]** | **7.6 [5.8 - 12.9]** | **11.9 [5.6 - 18.1]** | **10.1 [2.1 - 15.5]** | **0.0 [0.0 - 0.0]** | **13.8 [5.7 - 26.0]** |
| **Change, n (%)** | **85.5% (471)** | **72.0% (273)** | **70.3% (156)** | **61.2% (82)** | **49.3% (36)** | **51.6% (16)** | **66.7% (8)** | **75.0% (6)** | **83.3% (5)** | **20.0% (1)** | **0% (0)** | **74.1% (1054)** |
| ***By change reason, expressed as % of changers (n)*** | | |  |  |  |  |  |  |  |  |  |  |
| Remission | 36.3% (171) | 46.2% (126) | 39.1% (61) | 25.6% (21) | 22.2% (8) | 18.8% (3) | 12.5% (1) | 16.7% (1) | 20.0% (1) | 0% (0) | 0% (0) | 37.3% (393) |
| Inefficacy | 42.9% (202) | 34.1% (93) | 36.5% (57) | 51.2% (42) | 58.3% (21) | 56.3% (9) | 75.0% (6) | 83.3% (5) | 80.0% (4) | 100% (1) | 66.7% (2) | 41.7% (440) |
| Adverse event | 18.7% (88) | 14.7% (40) | 20.5% (32) | 17.1% (14) | 16.7% (6) | 12.5% (2) | 12.5% (1) | 0% (0) | 0% (0) | 0% (0) | 0% (0) | 17.4% (183) |
| Other | 2.1% (10) | 5.1% (14) | 3.8% (6) | 6.1% (5) | 2.8% (1) | 12.5% (2) | 0% (0) | 0% (0) | 0% (0) | 0% (0) | 0% (0) | 3.6% (38) |
| **No change, n (%)** | **14.5% (80)** | **28.0% (106)** | **29.7% (66)** | **38.8% (52)** | **50.7% (37)** | **48.4% (15)** | **33.3% (4)** | **25.0% (2)** | **16.7% (1)** | **80.0% (4)** | **100% (1)** | **25.9% (368)** |

**Table S2. Median time to event in months (with IQR) per change reason and treatment line.**

|  | **Remission** | **Inefficacy** | **Adverse event** |
| --- | --- | --- | --- |
| **Line 1** | 19.1 [13.6 - 25.9] | 5.5 [3.2 - 12.2]* | 8.2 [4.7 - 22.1] |
| **Line 2** | 20.4 [14.8 - 27.7] | 10.0 [4.1 - 28.3]* | 9.0 [2.4 - 16.4] |
| **Line 3** | 18.2 [12.2 - 25.1] | 8.7 [4.9 - 16.8] | 10.5 [4.3 - 21.4] |
| **Line 4** | 17.4 [9.8 - 24.8] | 6.8 [3.6 - 15.0] | 8.5 [2.3 - 25.1] |
| **Line 5** | 21.2 [18.0 - 40.1] | 9.5 [4.3 - 17.6] | 9.5 [1.7 - 26.0] |

*Treatment line 2 was significantly different from treatment line 1 (p <0.05, Wilcoxon rank test)

IQR = interquartile range

**Table S3. Percentages of patients consecutively experiencing the same change reason, compared to the total number of patients.**

| **Change reason** | **Line 1** | **Line 2** | **Line 3** | **Line 4** | **Line 5** |
| --- | --- | --- | --- | --- | --- |
| *Remission* | 31.0% (171/551) | 7.4%  (41/551) | 2.2%  (12/551) | 0.7%  (4/551) | 0%  (0/551) |
| *Inefficacy* | 36.7% (202/551) | 8.0%  (44/551) | 2.7%  (15/551) | 1.5%  (8/551) | 0.0%  (0/551) |
| *Adverse event* | 16.0% (88/551) | 1.5%  (8/551) | 0.2%  (1/551) | 0%  (0/551) | 0%  (0/551) |
